# Cardiothoracic Surgery Management of Giant Bullous Lung Disease Initially Misdiagnosed as Pneumothorax: A Case Report

**DOI:** 10.7759/cureus.36313

**Published:** 2023-03-17

**Authors:** Xuanzhen Piao, Fadi Alyass, Arshad Yousuf

**Affiliations:** 1 Radiology, Piedmont Macon, Macon, USA; 2 Internal Medicine, Piedmont Macon, Macon, USA; 3 Cardiothoracic Surgery, Piedmont Macon, Macon, USA

**Keywords:** emphysema, bullae, pneumothorax, giant bullous emphysema, vanishing lung syndrome

## Abstract

Giant bullous emphysema, also known as "vanishing lung syndrome", is a rare manifestation of chronic obstructive pulmonary disease (COPD) that is associated with high mortality. Cigarette smoking and alpha-1 antitrypsin deficiency (A1AD) are two main causes that result in permanent enlargement of airspaces, inefficient gas exchange, fibrosis of the airways, and collapse of the alveoli. A typical presentation can be found in a long-term smoker with dyspnea on exertion and progressive shortness of breath that may be associated with a productive cough. One of the clinical difficulties in diagnosing giant bullous emphysema is separating it from other etiologies like pneumothorax. It is paramount to differentiate giant bullous emphysema from pneumothorax as the management is completely different; both can, however, have similar clinical presentations and radiographic manifestations on initial assessment. In this report, we present the case of a 39-year-old African American male who presented with worsening shortness of breath and productive cough and was found to have bullous emphysema but was misdiagnosed and treated for pneumothorax in the initial encounter. The purpose of this case report is to raise awareness of this condition in the medical literature and discuss the similarity of bullous emphysema and pneumothorax in clinical presentation and radiographic findings, as well as the differences in treatment options.

## Introduction

Bullous emphysema is characterized by an air-filled bulla that is greater than 1 cm in diameter within the lung. It developed due to chronic inflammation of the distal airspaces that results in emphysematous destruction of the lung parenchyma, which subsequently leads to the breakdown of the alveolar wall and permanent enlargement of airspaces [1]. The two most common causes are cigarette smoking and alpha-1 antitrypsin deficiency (A1AD) [2]. Patients with this condition can be asymptomatic, but typically present with worsening dyspnea, productive cough, wheezing, respiratory failure, and occasionally spontaneous pneumothorax due to ruptured giant bullae [3]. The radiological criteria for giant bullous emphysema are described as bullae occupying at least one-third of the hemithorax and can be found in one or both upper lung lobes [4]. It is challenging but chiefly important to differentiate giant bullous emphysema from pneumothorax, as the immediate appropriate management differs and can prevent cataphoric complications. High-resolution computed tomography is the diagnostic imaging modality to help distinguish these two etiologies [2]. We present the case of a 39-year-old African American male who presented with worsening shortness of breath and productive cough and was found to have giant bullous emphysema but was misdiagnosed and treated for pneumothorax in the initial encounter.

## Case presentation

**Figure 1:**
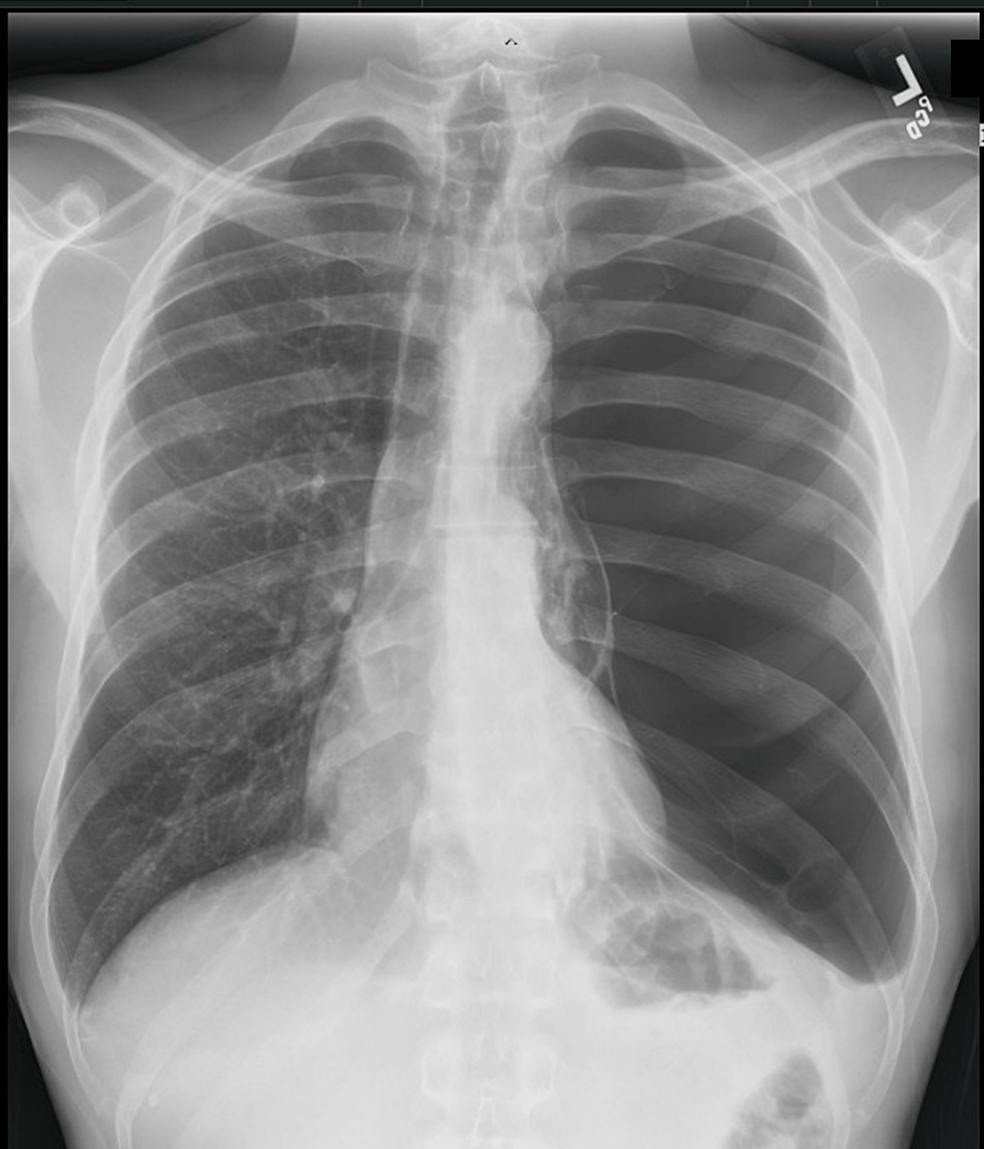
Initial plain film chest x-ray The initial plain film chest x-ray from the initial patient encounter at an outside hospital shows diffuse lucency throughout the left hemithorax. The right lung, however, appears well aerated without any confluent infiltrate or pneumothorax. This initial imaging may be confused with either a chronic pneumothorax or large bullae, given the patient's history and reasoning for the imaging.

A 39-year-old African American male with a past medical history of essential hypertension was transferred for medical management from an outside institution. The patient was misdiagnosed with pneumothorax and had a Fuhrman chest tube placed. On initial presentation at the outside facility, the patient presented with clinical symptoms of congestion, body aches, and a productive cough with thick but clear sputum and associated blood-tinged sputum for two weeks duration. The symptoms were unabated with self-treatment with over-the-counter cold and flu medication. The initial chest x-ray showed a left-sided large pneumothorax (Figure 1), and a Fuhrman chest tube was placed after consulting the hospitalist and cardiothoracic surgeon.

**Figure 2:**
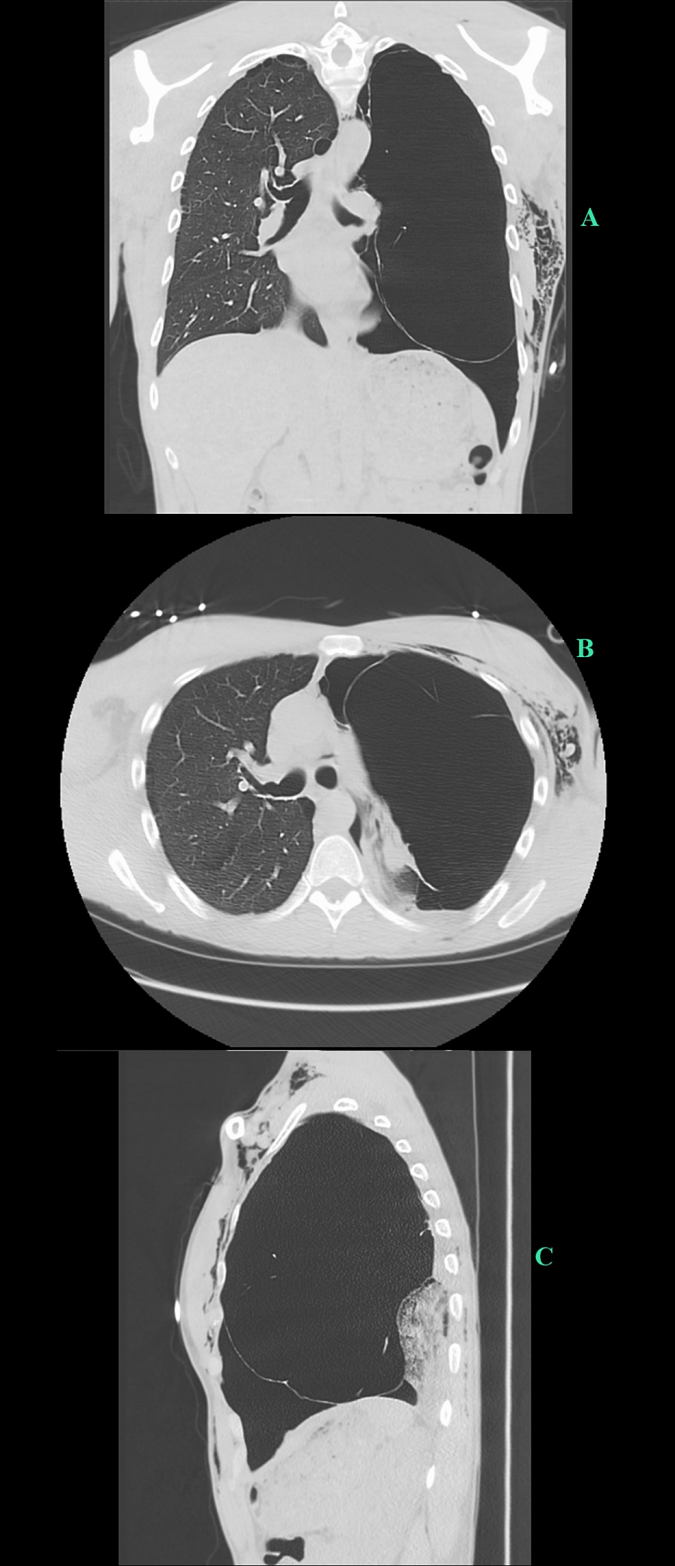
Chest computed tomography of giant bullae Chest CT showed large bullae comprising over 70% of the patient's left lung. It is seen in the coronal plane (A), axial plane (B), and sagittal plane (C). This is consistent with a severe bullous disease in the left hemithorax with dense compressive atelectasis of the left lung, which is best seen on the sagittal plane (C). The large bullae in the left lung measure 10.5 by 17.5 by 23.0 cm. There is also a superimposed pneumothorax in the left lung base.

The patient was admitted for inpatient management of a pneumothorax when a repeat chest x-ray did not show full expansion of the lung. Of note, the patient had subacute emphysema on the left side of the chest with a positive ginkgo leaf sign on the chest x-ray. Subsequently, a computed tomography (CT) of the patient’s chest was performed, and it revealed a large bullous disease with a small apical pneumothorax with significant blebs in the same region (Figure 2).

Cardiothoracic surgery services were consulted for management, which included embolectomy or lobectomy with or without pleurodesis. At the time, the patient was breathing room air and clinically stable, aside from a complaint of small-volume hemoptysis. At that point, the patient was transferred to a facility capable of providing higher-level care. Following the patient’s initial presentation and evaluation by cardiothoracic surgery, he underwent left video-assisted thoracoscopic surgery (VATS), multiple bullectomies, as well as mechanical and chemical pleurodesis with talc. During VATS, several large bullae arising from the left upper lobe were visualized, and they were removed with an endoscopic gastrointestinal anastomosis stapler. Mechanical pleurodesis was achieved using a Bovie electrocautery pad and talc over the visceral and parietal pleura, and a single chest tube was placed. The patient tolerated the procedure well at first, but the postoperative course was complicated by air leakage from the chest tube, which was later changed to a suction tube paired with a water seal that gradually decreased air leakage until the cessation of the leak. Serial X-rays every other day afterwards showed no pneumothorax or evidence of any air leaking. The chest tube was pulled, and the patient was discharged home; however, afterward, he was lost to follow-up.

## Discussion

Giant bullous emphysema, which was originally described by Burke in 1937, is a distinct clinical condition caused by chronic inflammation that results in emphysematous destruction of the lung parenchyma and eventually respiratory failure [5]. Giant bullous emphysema is commonly found in long-term smokers and patients with alpha-1-antitrypsin deficiency and is associated with a high mortality rate that affects greater than five percent of the population worldwide with a prevalence of 12% in adults over the age of 30 [1]. Therefore, it is vital to diagnose early and provide appropriate treatment. However, it is challenging to differentiate giant bullous emphysema from pneumothorax solely based on the clinical presentation and plain radiograph. As both conditions can present with cough, dyspnea, and progressive respiratory difficulties. In addition, on plain radiography, bullae appear as avascular radiolucent areas with a thin curvilinear wall that is usually less than 1mm in thickness [6]. Therefore, a chest x-ray is inadequate to reveal two etiologies. The gold standard diagnostic imaging modality is chest CT, which reveals multiple large bullae, ranging from 1 to 20 cm in diameter, without a single dominant giant bulla [7]. In contrast to pneumothorax, where needle aspiration or chest tube placement is the definitive treatment, the management of bullous emphysema is quite different. For asymptomatic patients, a relatively conservative approach, including nebulized bronchodilators, is employed [2]. However, in symptomatic patients, surgical interventions including stapled bullectomies, endocavitary drainage, volume reduction with video-assisted thoracoscopic surgery, one-way endobronchial valves, or lung transplants can be considered [2]. Regardless of the clinical presentation, the workup should include a pulmonary function test and serum alpha-1-antitrypsin level. This patient similarly underwent surgical interventions, and the symptom improvement can be measured by a pulmonary function test and subjective dyspnea scoring. Clinical improvement has been reported in 80%-100% of postoperative patients in numerous studies [8].

## Conclusions

Giant bullous emphysema should be differentiated from pneumothorax in an acute setting. Both conditions have similar clinical and radiologic presentations, which make a correct diagnosis difficult yet important for definitive treatment. A chest CT should be considered in all patients when considering the two diseases to differentiate them, as a plain-film chest x-ray is unreliable. The management of giant bullous emphysema differs from that of pneumothorax, in which needle aspiration or chest tube placement is not advised. Moreover, giant bullous emphysema patients require a conservative approach with nebulized bronchodilators or surgical interventions if symptomatic.

## References

[1] Siddiqui NA, Mansour MK, Nookala V (2022). Bullous Emphysema. https://www.ncbi.nlm.nih.gov/books/NBK537243/.

[2] Yousaf MN, Chan NN, Janvier A (2020). Vanishing lung syndrome: an idiopathic bullous emphysema mimicking pneumothorax. Cureus.

[3] Gao X, Wang H, Gou K (2015). Vanishing lung syndrome in one family: five cases with a 20-year follow-up. Mol Med Rep.

[4] Neviere R, Catto M, Bautin N, Robin S, Porte H, Desbordes J, Matran R (2006). Longitudinal changes in hyperinflation parameters and exercise capacity after giant bullous emphysema surgery. J Thorac Cardiovasc Surg.

[5] Burke RM (1937). Vanishing lungs: a case report of bullous emphysema. Radiology.

[6] Aramini B, Ruggiero C, Stefani A, Morandi U (2019). Giant bulla or pneumothorax: how to distinguish. Int J Surg Case Rep.

[7] Stern EJ, Webb WR, Weinacker A, Müller NL (1994). Idiopathic giant bullous emphysema (vanishing lung syndrome): imaging findings in nine patients. AJR Am J Roentgenol.

[8] van Berkel V, Kuo E, Meyers BF (2010). Pneumothorax, bullous disease, and emphysema. Surg Clin North Am.

